# Body mass index and waist-to-hip ratio misclassification of overweight and obesity in Chinese military personnel

**DOI:** 10.1186/s40101-020-00236-8

**Published:** 2020-08-24

**Authors:** Qingqing Zhu, Binbin Huang, Qiaoli Li, Liqian Huang, Wenbo Shu, Lin Xu, Qiongying Deng, Ziliang Ye, Chunyan Li, Peng Liu

**Affiliations:** 1 Guangxi Medical University, Nanning, 530021 Guangxi China; 2 Department of Anatomy, Guangxi Medical University, Nanning, 530021 Guangxi China; 3 The First Affiliated Hospital of Guangxi Medical University, Nanning, 530021 Guangxi China

**Keywords:** Chinese military personnel, Obesity misclassification, Body composition, BMI, BFP, WHR

## Abstract

**Background:**

The rising prevalence of obesity in military personnel has raised great concerns. Previous studies suggest that body mass index (BMI)- and waist-to-hip ratio (WHR)-based obesity classifications in US military personnel and firefighters have high false negative and subsequently cause obesity misclassification.

**Objective:**

To determine whether BMI and WHR could reflect the fat mass of Chinese military personnel.

**Methods:**

Three hundred fifty-three male Chinese military personnel and 380 age-matched male adults were recruited. Obesity classification was defined by BMI, WHR, and body fat percentage (BFP).

**Results:**

Chinese military personnel had extremely low obesity rate determined by either BFP (0.3%) or BMI (0.6%). By combining overweight and obese individuals, BMI- and WHR-determined prevalence of overweight/obesity was 22.4% and 17.0% compared to BFP-based standard (4.0%) (*P* < 0.05). In reference to BFP, BMI and WHR have high false-positive rate compared to the control group. Further analysis showed that Chinese military personnel consisted of high percentage of BFP^low^BMI^high^ and/or BFP^low^WHR^high^ subpopulations. Eighty-one percent of BMI^high^ and 78.3% of WHR^high^ of them were BFP low.

**Conclusions:**

Chinese military personnel has extremely low obesity rate. BMI and WHR have high false-positive rates in reference to BFP, which cannot accurately reflect the mass of adipose tissue and leads to obesity misclassification.

## Background

The rising prevalence of obesity remains a growing public concern, and it is considered the fifth most important risk factor contributing to global death [1] Obesity and overweight lead to multiple health dysfunctions including cardiovascular diseases, cancers, and metabolic syndromes as well as increased risk of mortality [2, 3]. Unfortunately, military personnel worldwide are not immune to this obesity epidemic. A study analyzing the trend in overweight and obesity from 1989 to 2012 among the US army revealed that the prevalence of obesity increased from 5.6% in 1989 to 8.0% in 2012 and peaked at 12.3% in 2009 [4]. The body mass index (BMI)-defined obesity prevalence in the British army was 12% in 2014, while a higher percentage of males were obese [5]. The consequence of overweight and obesity are costly to the military in terms of medical and related financial burdens. Excess weight has been associated with numerous health issues for the military personnel such as hypertension, diabetes, sleep apnea [6], and musculoskeletal injuries [7, 8]. In particular, musculoskeletal conditions together with overweight significantly increases the risk of disabilities in the US army which leads to the increased odds of disability discharge [9, 10].

To maintain the physical fitness of the fighting force, military personnel mostly is subjected to active and intense military trainings [11], which contribute to the changes of individual body composition. Individuals in the structural physical training programs of either US or Italian Army experienced beneficial changes of body composition, along with body fat reduction and muscle mass increase [12, 13]. Of note, the effects of military training on body composition vary by different factors, such as age, body-shape [14], and length of military service [15].

Body composition quantifies body mass into basic components at different levels, and its measurement at the tissue level accesses the contribution of specific tissues to body mass: skeletal muscle, adipose, bone, blood, viscera, and brain [16]. The body fat percentage (BFP) is a commonly used index for obesity classification and calculated based on the total mass of fat measured at the tissue level. BMI and waist-to-hip ratio (WHR) are anthropometric indicators representing the body composition accessed at the whole-body level, which focus on the body size, shape, physique, and proportions [16–18]. Using BMI and WHR to classify obesity for military personnel as well as individuals in physical-active occupational groups, such as police officers or firefighters, has raised concerns regarding how well it reflects body composition, specifically about distinguishing lean and fat mass, because these populations may have greater muscle mass [19–23]. In a study evaluating active duty US military personnel, for instance, in BF%-defined obese men, 35% and 42% were misclassified as non-obese (false negatives) using BMI and circumference methods, respectively [19]. Similar higher false-negative rates of BMI-defined obesity has been shown in a US police officer study [20] and US firefighter cohort studies [21, 23].

Unlike Caucasians, the body composition of people living in the East and Southeast Asia areas is feathered with low BMI but high BFP as well as higher prevalence of overall or abdominal obesity [24, 25], which lead to an obesity misclassification that a BMI-normal individual could be BFP-defined obese [26, 27]. A previous cross-sectional study in Singapore suggested that BMI method under-predicted BFP-defined obesity for all three different ethnic groups, Chinese, Malays, and Indians [28]. Therefore, for Asian people, a reasonable BMI-cut-off point is important [28, 29]. Most previous studies involved individuals served in the US or European armies to determine whether BMI and WHR can well reflect obesity, while such knowledge is limited for Asian military personnel with different ethnic physical characteristics. So in this current study, we purposed to evaluate the accuracy of BMI and WHR to estimate obesity status in Asian military personnel based on the criteria of BFP. We recruited military personnel serving in the Chinese army as well as age-matched local residents and compared and evaluated different methods (BMI, WHR, and BFP) in obesity classification.

## Material and methods

### Subjects

In this cross-sectional study, a total of 969 male military personnel aged 18–25 years and 1912 age-matched male local residents living in the subtropical regions of Guangxi Zhuang Autonomous Region, China, were randomly recruited. The study protocol was approved by the Ethics Committee of Guangxi Medical University. Informed consent was taken from each participant. Active smokers and participants with metabolic dysfunction, secondary obesity, malignant tumor, pharmacological treatments, and surgery in the past year were excluded. According to the inclusion and exclusion criteria, 353 male military personnel and 380 male age-matched adults were finally included.

### Basic demographic data

Basic demographic variables including height, weight, fat mass, fat free mass, trunk fat mass, trunk fat percentage, visceral fat mass, subcutaneous fat mass, left arm fat mass, left arm fat percentage, left leg fat mass, left leg fat percentage, right arm fat mass, right arm fat percentage, right leg fat mass , right leg fat percentage, muscle mass, trunk muscle mass, left arm muscle mass, left leg muscle mass, right arm muscle mass, right leg muscle mass, BMI, BFP, and WHR were recruited.

### Measurement of physical parameter assessment

Body composition indices were measured using bioelectrical impedance analysis (BIA) method by the body composition analyzer MC-180 (TANITA, Japan) including the body composition in segmental parts of the whole body including four limbs and trunk area. Body compositions were measured in the same well-ventilated room with controlled temperature and humidity, while the subjects were instructed to follow standard food and fluid protocol to be rested, overnight fasted, and hydrated state, empty their bladder and bowel before the measurement, avoid strenuous exercise, alcohol, stimulants, or depressants for 24 h before testing, with only light clothing, after removing shoes, socks, and all metal accessories [30, 31]. The impedance instrument used in this study has been validated to predict body composition consistent with DXA in previous overweight and obese adolescents [32].

The classification of BMI, BFP, and WHR were based on the following standards [33]:

- BMI-based criteria: underweight (BMI < 18.5), normal weight (18.5 ≤ BMI < 24), overweight (24 ≤ BMI < 28), and obese (BMI ≥ 28), which were in accordance with the recommendation defined by Working Group on Obesity in China [34, 35]. In indicated analyses, BMI-underweight and BMI-normal weight were combined and referred as BMI-low group; BMI-overweight and BMI-obese were combined and referred as BMI-high group.
- BFP-based criteria: low fat (< 11%), normal fat (11% ≤ BFP < 22%), overweight (22% ≤ BFP <27%), and obese (BFP≥27%), which was consistent with the cut-off value of previous study [33]. The cut-off point 22% was selected to differentiate low BFP (BFP < 22%) and high BFP (BFP ≥ 22%) groups.
- WHR-based criteria: low WHR (WHR < 0.91) and high WHR (obese, WHR ≥ 0.91) according to the recommended cut-off value of previous study [33].

Previous studies use BFP as gold standard to estimate accuracy of obesity variables to measure obesity status [36–38]. So, in this study, BFP was set as referenced standard of defining obesity to evaluate the accuracy of BMI and WHR indexes to distinguish obesity status in Chinese military personnel and healthy controls.

### Statistical analysis

Statistical analysis was performed using the SPSS 20.0 software. Statistical significance was set at *P* < 0.05. Statistical significance of basic characteristic was assessed using Student *t* test. Subjects were classified based on definition of BMI, WHR, and BFP categories. Chi-square test was used to compare the different obesity classifications defined by above BMI, BFP, and WHR categories, and results were exhibited by bar diagrams and scatter plots.

## Results

The characteristics of the participants, consisting of 353 military personnel and 380 of age-matched male adults were summarized in Table 1. The means of height, weight, BMI, WHR, fat mass, fat free mass, and muscle mass were significantly higher in the military personnel group than in the control group. The difference in fat distribution was observed in subcutaneous fat mass but not in visceral fat mass. Military personnel had more left/right arm fat and muscle mass, left/right leg fat and muscle mass, and left/right leg fat percentage respectively (*P* < 0.05). On the contrary, there was no significant difference in age, trunk fat mass, trunk fat percentage, and body fat percentage between the military personnel and control groups.

**Table 1:** Comparison of physical parameters between military personnel and age-matched control adults

Item	Military personnel (*n* = 353)		Control group (*n* = 380)		*P* value
	Mean	SD	Mean	SD	
Age (years)	20.14	1.42	20.00	1.44	0.17
Height (cm)	170.98	5.05	168.89	5.98	< 0.01
Weight (kg)	65.16	7.73	58.59	8.93	< 0.01
Fat mass (kg)	8.98	4.16	7.88	4.70	< 0.01
Fat-free mass (kg)	56.20	4.77	50.72	5.43	< 0.01
Trunk fat mass (kg)	4.36	2.52	4.14	2.82	0.27
Trunk fat percentage (%)	12.92	6.12	13.27	6.72	0.47
Visceral fat mass (kg)	0.96	0.77	0.86	0.87	0.08
Subcutaneous fat mass (kg)	8.01	3.42	7.02	3.82	< 0.01
Left arm fat mass (kg)	0.43	0.43	0.35	0.21	< 0.01
Left arm fat percentage (%)	10.56	3.77	10.29	4.33	0.38
Left leg fat mass (kg)	1.95	0.70	1.58	0.80	< 0.01
Left leg fat percentage (%)	14.75	3.86	13.01	4.62	< 0.01
Right arm fat mass (kg)	0.37	0.16	0.33	0.17	< 0.05
Right arm fat percentage (%)	9.85	3.59	9.60	4.17	0.39
Right leg fat mass (kg)	1.98	0.71	1.59	0.81	< 0.01
Right leg fat percentage (%)	14.77	3.73	12.89	4.63	< 0.01
Muscle mass (kg)	53.29	4.53	48.08	5.16	< 0.01
Trunk muscle mass (kg)	26.70	2.79	24.02	2.72	< 0.01
Left arm muscle mass (kg)	3.06	2.29	2.53	1.04	< 0.01
Left leg muscle mass (kg)	10.28	1.02	9.45	1.18	< 0.01
Right arm muscle mass (kg)	2.91	0.31	2.56	0.30	< 0.01
Right leg muscle mass (kg)	10.44	1.04	9.62	1.17	< 0.01
BMI (kg/m^2^)	22.26	2.28	20.50	2.67	< 0.01
BFP (%)	13.35	4.75	12.80	5.51	0.15
WHR	0.87	0.03	0.86	0.04	< 0.01

Note: *BMI* body mass index, *BFP* body fat percentage, *WHR* waist-to-hip ratio

Since the means of BMI and WHR but not BFP were higher in military personnel compared to control adults, we next determined the obesity prevalence defined by different methods; BMI, WHR, and BFP. BMI, WHR, and BFP classifications were shown in Table 2. 0.3% of military personnel but 2.4% of control adults were obese based on BFP criteria, respectively (*P* < 0.05). Consistently, 0.6% of military personnel and 2.1% of control adults were BMI-defined obese, but it showed no significant statistical significance. It is worthy to note that only 1 out of 353 military personnel was BFP-defined obese and 2 were BMI-defined obese (Table 2). These results indicated that the prevalence of BFP- and BMI-defined obesity were extremely low for Chinese military personnel.

**Table 2:** BMI-, BFP-, and WHR-defined overweight and obesity prevalence in Chinese military personnel compared to age-matched control adults

Obesity criteria	Obesity classified by BFP		Obesity classified by BMI		Overweight and obesity classified by BFP		Overweight and obesity classified by BMI		Obesity classified by WHR	
	No	Yes	No	Yes	No	Yes	No	Yes	No	Yes
Military personnel	352 (99.7)	1* (0.3)	351 (99.4)	2 (0.6)	339 (96.0)	14* (4.0)	274 (77.6)	79** (22.4)	293 (83.0)	60* (17.0)
Control group	371 (97.6)	9 (2.4)	372 (97.9)	8 (2.1)	350 (92.1)	30 (7.9)	340 (89.5)	40 (10.5)	335 (88.2)	45 (11.8)

Note: *BMI* body mass index, *BFP* body fat percentage, *WHR* waist-to-hip ratio

*P < 0.05, **P < 0.01, with statistical significance. The number in the parenthesis represents the percentage of each subpopulation in ratio to the total populations in military personnel and control group, respectively. That is *n* (%)

Because of the limited case numbers for both BMI-obese group and BFP-obese group, we combined overweight group and obese group together for further analysis. After combining, the prevalence of BFP-defined obesity and overweight was 4.0% and 7.9% for the military personnel and the control group, respectively (*P* < 0.05) (Table 2). On the contrary, 22.4% of military personnel and 10.5% of the control adults were BMI-defined overweight and obese (*P* < 0.01). The prevalence of WHR defining obesity indicated 17.0% in military personnel and 11.8% in control adults (*P* < 0.05). Taken together, these results suggest that the prevalence of overweight and obesity for military personnel were lower than control adults using BFP, BMI, and WHR obesity classification.

**Fig. 1:**
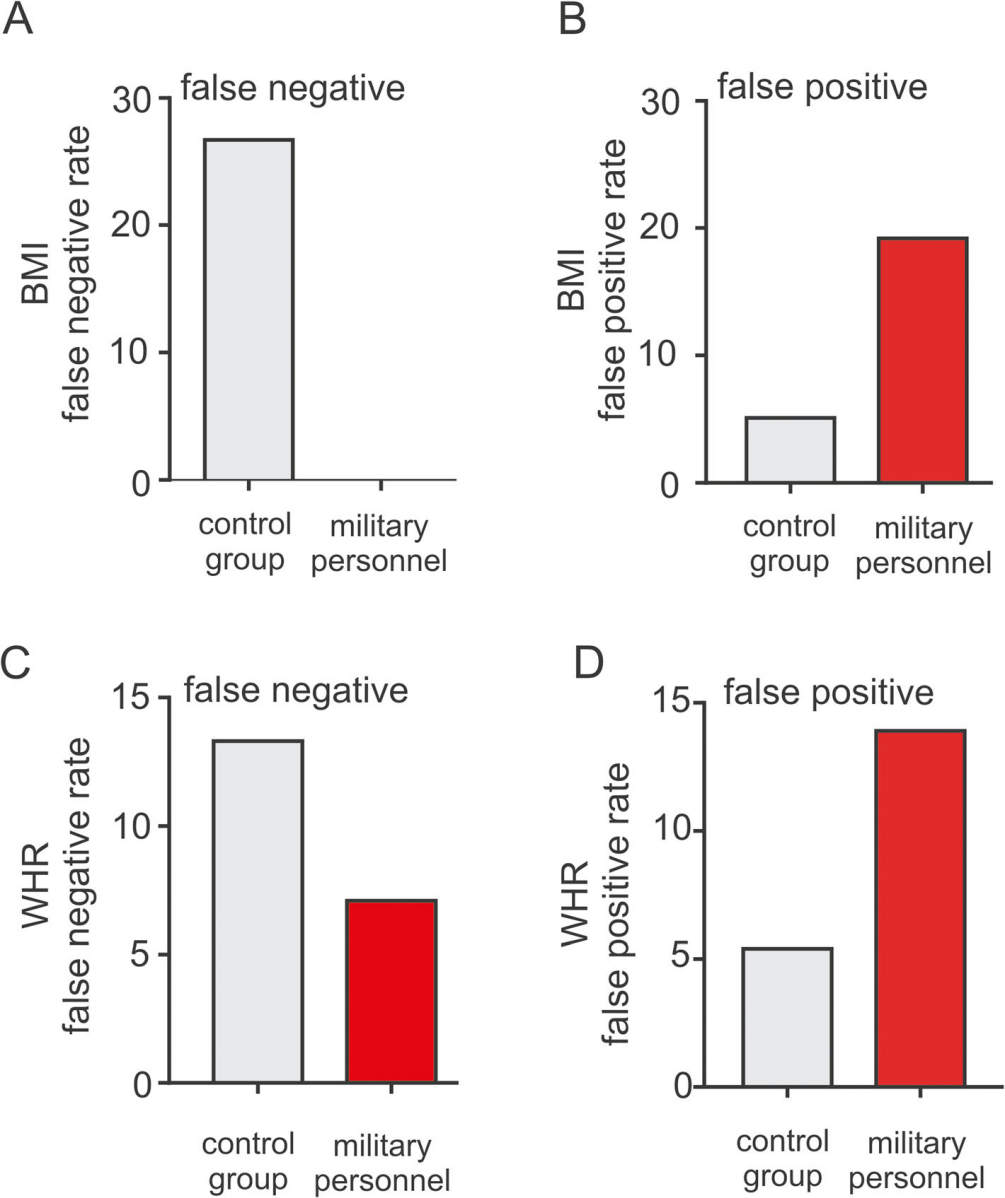
False negative (**a**, **c**) and positive (**b**, **d**) rates for BMI and WHR method in obesity classification with reference to BFP between male military personnel and control groups. Note: BMI, body mass index; WHR, waist-to-hip ratio; BFP, body fat percentage. False-positive rates were calculated using following equations: False-positive rate = BMI or WHR ^overweight/obese^ BFP-^nonoverweight/nonobese^/BFP^nonoverweight/nonobese^. False-negative rate = BMI or WHR ^nonoverweight/non obese^ BFP ^overweight/obese^/BFP^overweight/obese^

Table 3 suggested that BMI and WHR criteria was inclined to increase the prevalence of overweight and obesity for military personnel in reference to BFP-based obesity classification (*P* < 0.01). And previous studies have shown that BMI and WHR methods had high false-negative rates in reference to BFP-defined obesity for US military personnel and firefighters [19, 21, 23]. So, we examined the false-positive and false-negative rates of BMI- and WHR-determined overweight/obesity in reference to BFP-defined overweight/obesity as shown in Table 3 and Fig. 1. Unlike previous US studies, for Chinese military personnel, approximately 19.2% and 13.9% of BFP-defined non-overweight/non-obese individuals were misclassified as BMI-and WHR-defined overweight/obese (false positive), which were much higher than the control group (5.1% for BMI and 5.4% for WHR). On the contrary, the false-negative rates of BMI- and WHR-defined overweight/obesity for military personal were much lower than the control group: 0% and 7.1% of BFP-obese military personnel were BMI- and WHR-defined non-overweight/non-obese, compared to 26.7% and 13.3% of BFP-overweight/obese for control adults. Taken together, these results suggest that BMI and WHR cause high rates of false-positive in reference to BFP-defined overweight/obesity for Chinese military personnel.

**Table 3:** Rates of false positives or negatives of BMI- and WHR-defined obesity based on the criteria of BFP

Obesity status	BFP standard^a^		*P* value		Obesity status	BFP standard^a^		*P* value	
	Overweight/obese (BFP ≥ 22%)	Non-overweight/non-obese (BFP < 22%)		Rates of false positive or negative		Overweight/obese (BFP ≥ 22%)	Non-overweight/non-obese (BFP < 22%)		Rates of false positive or negative
Military personnel									
Overweight/obese (BMI ≥ 24 kg/m2)	14	65	< 0.01	False positive 19.2% (65/339)	Obese (WHR ≥ 0.91)	13	47	< 0.01	False positive 13.9% (47/339)
Non-overweight/non-obese (BMI < 24 (kg/m2)	0	274		False negative 0% (0/14)	Non-obese (WHR < 0.91)	1	292		False negative 7.1% (1/14)
Age-matched control group									
Overweight/obese (BMI ≥ 24 (kg/m2)	22	18	0.076	False positive 5.1% (18/350)	Obese (WHR ≥ 0.91)	26	19	< 0.01	False positive 5.4% (19/350)
Non-overweight/non-obese (BMI < 24 (kg/m2)	8	332		False negative 26.7% (8/30)	Non-obese (WHR < 0.91)	4	331		False negative 13.3% (4/30)

Note: *BMI* body mass index, *BFP* body fat percentage, *WHR* waist-to-hip ratio

^a^For each analysis, BFP served as standard and BMI or WHR-based categories as “screening tests”

False-positive and false-negative rates were calculated using following equations: False positive rate = BMI or WHR^overweight/obese^ BFP^nonoverweight/nonobese^/BFP ^nonoverweight/nonobese^. False negative rate = BMI or WHR^nonoverweight/non obese^ BFP^overweight/obese^/BFP^overweight/obese^. The numbers in the parenthesis were represented according to the formulas of calculating false positive/negative rates above

**Fig. 2:**
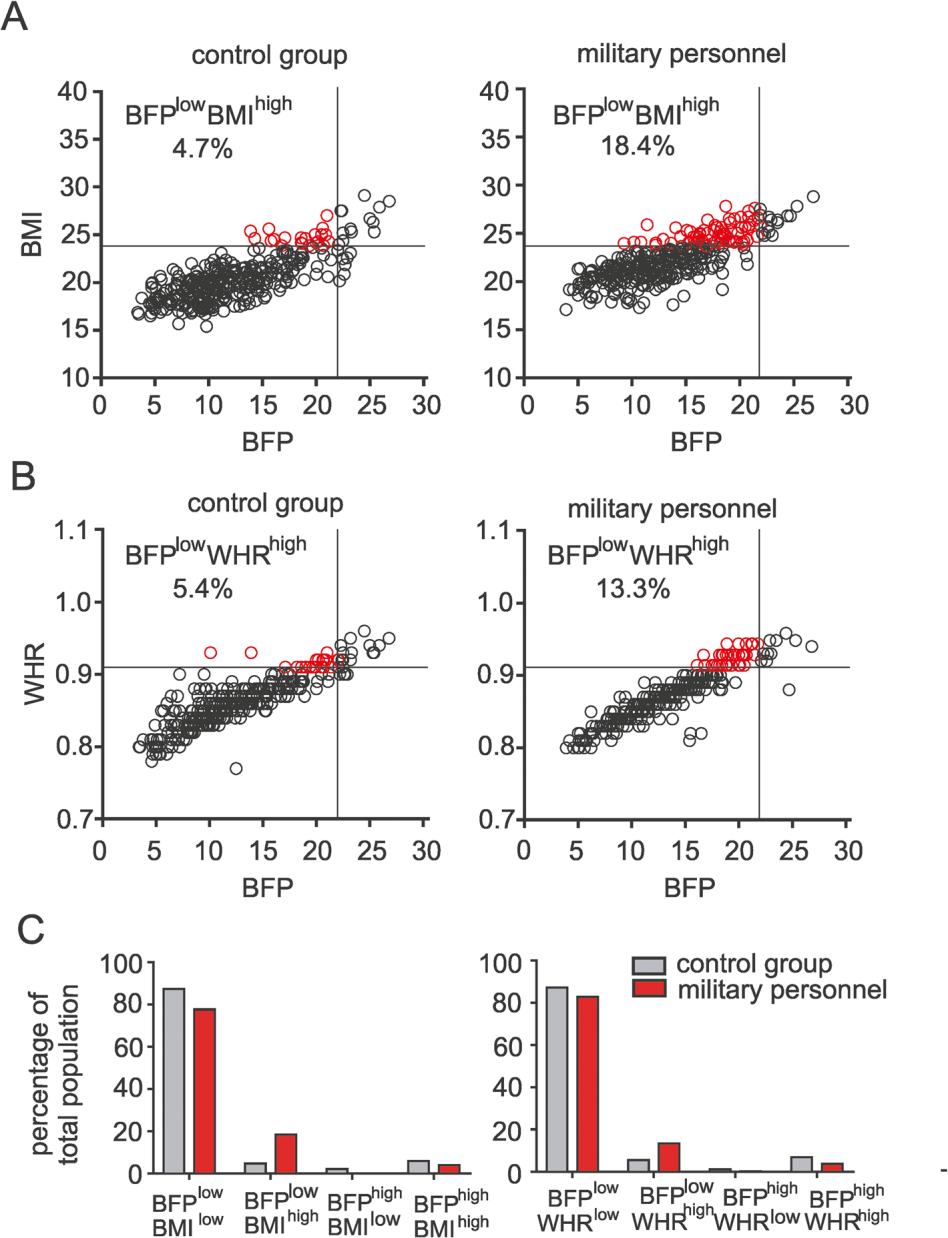
The distribution of BFP/BMI (**a**, **b**) and BFP/WHR (**c**, **d**) dual-criteria classified subpopulations between male military personnel and control groups. Note: The percentage of BFP^low or high^BMI^low or high^ and BFP ^low or high^WHR^low or high^ were calculated as the number of BFP^low or high^BMI^low or high^ and BFP ^low or high^WHR^low or high^ to the number of total population

To elucidate the above disagreement, we next divided military personnel and control groups into dual criteria-based subgroups: BMI/BFP (Table 4) and WHR/BFP (Table 5). As shown in Tables 4, 5 and Fig. 2, military personnel had a higher percentage of BFP^low^BMI^high^ (18.4% vs 4.7%) and BFP^low^WHR^high^ (13.3% vs 5.0%) subpopulations ratio to total populations in comparison with control adults. Whereas the ratios of BFP^high^BMI^low^ (0% vs 2.1%) and BFP^high^WHR^low^ (0.3% vs 1.1%) subgroups were extremely lower in military personnel than control individuals. We further analyzed the percentage of BFP^low^ subpopulations within either BMI^high^ or WHR^high^ groups. Consistently, 81.0% of military personnel but 45.0% of control adults were BFP^low^ (Table 6) within the BMI^high^ populations. Within WHR^high^ group, the percentages of BFP^low^ subpopulations were 78.3% and 42.4% for military personnel and control adults, respectively. These results suggest that BMI and WHR were inaccurate to reflect body fat mass for Chinese military personnel, which contribute to the obesity misclassification.

**Table 4:** BMI-/BFP-criteria based obesity classification

Item	BMI: *n*(%)			*P* value
	Low BMI	High BMI	Total	
Military personnel				
Low BFP	274 (77.6)	65 (18.4)	339 (96.0)	< 0.01
High BFP	0 (0.0)	14 (4.0)	79 (4.0)	
Control group				
Low BFP	332 (87.4)	18 (4.7)	350 (92.1)	< 0.01
High BFP	8 (2.1)	22 (5.8)	30 (7.9)	

Note: *BMI* body mass index, *BFP* body fat percentage, *WHR* waist-to-hip ratio. *Low BFP* population included low-fat and normal-fat individuals, *High BFP* population included overweight and obese individuals (cut-off point: 22%). *Low BMI* population included underweight and normal-weight individuals, *High BMI* population included overweight and obese individuals (cut-off point: 24 kg/m^2^). The number in the parenthesis represents the percentage of subpopulation in ratio to the total populations in military personnel and control group, respectively

**Table 5:** WHR-/BFP-criteria based obesity classification

Item	WHR: *n* (%)			*P* value
	Low WHR	High WHR	Total	
Military personnel				
Low BFP	292(82.7)	47 (13.3)	339 (96.0)	< 0.01
High BFP	1 (0.3)	13(3.7)	14 (4.0)	
Control group				
Low BFP	331 (87.1)	19 (5.0)	350 (92.1)	< 0.01
High BFP	4 (1.1)	26(6.8)	30 (7.9)	

Note: *BMI* body mass index, *BFP* body fat percentage, *WHR* waist-to-hip ratio. *Low BFP* population included low-fat and normal-fat individuals, *High BFP* population included overweight and obese individuals (cut-off point: 22%). *Low WHR* population included normal WHR individuals, *High WHR* population included obese individuals (cut-off point: 0.91)

**Table 6:** Number and percentage of BFP/BMI and BFP/WHR ratio to its subpopulations

Item	Total number	Ratio to low BFP population	Ratio to high BFP population	Ratio to high BMI population	Ratio to high WHR population
Military personnel					
Total number	/	339	14	79	60
BFP^low^/BMI^high^	65	65/339 (19.2%)	/	65/79 (81.0%)	/
BFP^low^/WHR^high^	47	47/339 (13.9%)	/	/	47/60 (78.3%)
BFP^high^/BMI^high^	14	/	14/14 (100%)	14/79 (17.7%)	/
BFP^high^/WHR^high^	13	/	13/14 (92.9%)	/	13/60 (21.7%)
Control groups					
Total number	/	350	30	40	45
BFP^low^/BMI^high^	18	18/350 (5.1%)	/	18/40 (45.0%)	/
BFP^low^/WHR^high^	19	19/350 (5.4%)		/	19/45 (42.2%)
BFP^high^/BMI^high^	22	/	22/30 (73.3%)	42/60 (55.0%)	/
BFP^high^/WHR^high^	26	/	26/30 (86.7%)	/	33/40 (57.8%)

Note: *BMI* body mass index, *BFP* body fat percentage, *WHR* waist-to-hip ratio. *BFP*^*low*^ population included low-fat and normal-fat individuals, *BFP*^*high*^ population included high-fat and obese individuals. *BMI*^*low*^ population included underweight and normal-weight individuals, *BMI*^*high*^ population included overweight and obese individuals. *WHR*^*low*^ population included normal WHR individuals, *WHR*^*high*^ population included obese individuals

## Discussion

Being paralleled with the rising prevalence of obesity globally, a worsening prevalence of obesity in military personnel has remained a growing concern as well [39, 40].A previous study reported that, for duty-active US military personnel, the prevalence of obesity has been doubled and reached to 20% in 2007/2008 compared to that of 10% in 2001/2003 [6]. Because weight or fat reduction among military personnel and attainment of desired body composition are considered important for physical appearance and military performance, it is essential to precisely assess the body composition for individual military personnel [11, 41, 42]. Previous studies focusing on US military personnel and firefighters suggested that either BMI or WHR method fails to reflect body fat accurately, while both BMI and WHR methods lead to the obesity misclassification with high false-negative rates in reference to BFP method [19, 21, 23]. However, it is uncertain whether such obesity misclassification is applicable for Asian military personnel, considering the difference of body composition between Caucasian and Asian populations [24, 25]. To answer this question, we recruited 353 male duty-active Chinese military personnel and 380 male age-matched local residents, measured their body compositions, and compared the obesity classification defined by different methods BMI, WHR, and BFP. Compared to age-matched male adult control group, the military personnel showed higher means of BMI and WHR as well as subcutaneous fat mass and muscle mass. No significant difference was noticed for BFP. The prevalence of BFP-(0.3%) and BMI-defined (0.6%) obesity was extremely low for Chinese military personnel, and only 1 out of 353 of soldiers was BFP-defined obese. With the respect to the reality of limited obesity case numbers, we combined obese and overweight individuals together. After combining, both BMI and WHR method have high rates of obese/overweight in reference to BFP method in the Chinese military personnel, along with high false-positive rate but low false-negative rates. Further analysis indicated that military personnel consisted of a higher percentage of BFP^low^BMI^high^ and/or BFP^low^WHR^high^ subpopulation in comparison with the control groups, which accounted for the disagreement of the obesity/overweight prevalence between BMI-/WHR- and BFP-defined obesity. We also found that most BMI- and WHR-high military personnel were BFP low. Taken together, our current study suggested that BMI and WHR have limited diagnostic accuracy to determine the obesity/overweight for military personnel. BFP alone or in combination with BMI and WHR might be reliable to assess the prevalence of obesity, while BMI and WHR could be more suitable to assess the physical shape for military personnel.

Among the commonly employed criteria used for obesity classification, BMI is cheap and convenient. However, BMI is limited to discriminate between fat mass, lean mass, and fat distribution, as BFP does [43, 44]. Especially, BMI tends to underestimate obesity prevalence defined by abnormal or excessive body fat accumulation, particularly in overweight individuals [45–47]. Excess body fat is the primary defining characteristic of obesity, and a precise measurement of the BFP is considered the reference method for defining obesity. Unlike BMI and BFP methods that are generally used to determine systemic obesity, WHR is a reference method to determine the visceral fat mass accumulation in the abdominal region of the body [48], and distinguish abdominal obesity from merely lower-body/peripheral obesity [49, 50]. It is well known that body composition, the relationship between BMI, WHR, and BFP, and their ideal cut-off points are ethnic groups, age, and sex-dependent [28, 51, 52]. So the ideal method for measuring obesity ought to be population-specific. For instance, both BMI and WHR have strong correlations with BFP for Turkish teenagers, while the area under the curve result suggests BMI is more useful than WHR to predict overweight or obesity [48]. On the contrary, a similar cross-sectional study in Vietnam adolescents suggested that BFP rather BMI provides a more accurate obesity assessment because BFP but not BMI was strongly correlated with fat content [43]. Therefore, the inconsistence by different obesity-defining methods results in the obesity misclassification.

The BMI-specific obesity misclassification is more common in overweight muscular individuals. There existed studies indicated that high level of BMI in adolescent athletes were more associated with higher levels of muscle and lean mass rather than adiposity [53, 54]. As for WHR, it showed lower screening performance and has high false-positive rates at distinguishing regional fat distribution, abdominal visceral fat or subcutaneous fat mass [55, 56]. Previous studies about US military personnel, firefighters, and policemen showed that BMI usually underestimated the prevalence of obesity along with high false-negative rate [19–23]. Because US military personnel and firefighters tends to have excess fat rather than body weight, their obesity was described as “skinny fat” obesity which may contribute to the high false-negative rate for BMI [19, 21]. However, the sampling strategies may affect the BMI accuracy as well. A previous study showed BMI had high false-positive rate in determining obesity of law enforcement police officers without any health issues [57]. On the contrary, another study including all police officers regardless of the health problem from smaller community departments showed high false-negative rate instead for BMI [20]. Such paradox was also observed for Chinese military personnel. After considering the limit cases of obesity in Chinese military personnel and combining obese/overweight individuals for analysis, we found that BMI and WHR have high false-positive rates in determining the prevalence of obesity/overweight in reference to BFP. Of note, it is hard to compare results from US military personals and occupational groups with our current study because the obesity rate is extremely low for our Chinese military personnel (0.3% and 0.6% BFP and BMI defined-obesity) compared to the participants in US studies. US military personnel or armed firefighter has higher prevalence of BFP-defined obesity which is about 10% [58, 59]. Furthermore, the body compositions may vary between Chinese and American populations which makes impossible to compare the above two populations directly [60, 61].

Our results (Tables 4 and 5) showed higher percentage of BFP^low^BMI^high^ and BFP^low^WHR^high^ subpopulations ratio to total populations in Chinese military personnel in comparison with control adults. Then, we carried out further analysis on these BMI/WHR^high^BFP^low^ subgroups. Importantly, within BMI/WHR ^high^ groups, about 80% of either BMI- or WHR-high Chinese soldiers were BFP low (Table 6), suggesting that BMI and WHR did not reflect the real fat content for most BMI^high^ and WHR^high^ military personnel. For military personnel, high protein diet and intensive military training have positive effects on the improvement of fat-free mass and skeletal muscle [62, 63]. A Finland study also showed increased lean tissue was observed in soldiers through military service [14]. We also found that our Chinese military personnel having significantly higher fat free mass and muscle mass than the control group. Therefore, it is plausible that high levels of muscle mass and fa-free mass in Chinese military personnel lead to higher percentage of BFP^low^BMI^high^ subpopulations and subsequently account for the BMI-determined high false-positive rate. WHR-specific obesity misclassification with high false-positive rate is probably due to the fact that Asians have more back muscle and upper-body subcutaneous fat but not visceral adiposity [64].

Another possible reason for the high false-positive rates is the setting of the cut-off point. In this study, the adopted BMI cut-off point was below the WHO standard because Asian adults are featured with less muscle mass but higher fat content [65–68]. In another word, a BMI-low Asian individual could be BFP-high. Therefore, the BMI cut-off points we used herein that below the WHO standard can increased the sensitivity in obesity classification but also contribute to the high false-positive rates for Chinese military personnel.

There are some limitations existed in this study. Firstly, this research is a cross-sectional study, and we have not carried out a follow-up for military personnel and controls. So the obesity status classified by BMI, BFP, and WHR fail to predict their prognosis and clinical outcomes, such as cardiovascular disease and metabolism syndrome, which worth our future investigation. Secondly, our study mainly focus on the obesity classification of young male military personnel, female or older military personnel are not included in our investigation, so we cannot provide guidance for researchers to select appropriate obesity indexes to reflect fat mass in female and older military personnel. In addition, we just recruit Chinese military populations for obesity analysis, which may not totally accurate to apply to other Asian, US, or European crowds. It is a good direction needs our further research.

## Conclusions

In summary, we concluded that BMI and WHR overestimated the prevalence of obesity and overweight for Chinese military personnel in reference to BFP. For Chinese military personnel, BMI-specific obesity misclassification was probably due to with the high levels of muscle mass and fat-free mass, while increased subcutaneous fat mass may contribute to WHR-specific obesity misclassification. BFP alone or in combination with BMI and WHR could be reliable to classify obesity for Chinese military personnel.

## Data Availability

The datasets used during our current study are available from the corresponding authors on reasonable request.

## Abbreviations

BMI: Body mass index
BFP: Body fat percentage
WHR: Waist-to-hip ratio
